# Cloning of Wing-Development-Related Genes and mRNA Expression Under Heat Stress in Chlorpyrifos-Resistant and -Susceptible *Plutella xylostella*

**DOI:** 10.1038/s41598-018-33315-z

**Published:** 2018-10-15

**Authors:** Xue Zhun Chen, Qi Xing Hu, Qi Qing Liu, Gang Wu

**Affiliations:** Key Laboratory of Biopesticide and Chemical Biology (Ministry of Education), Fujian Agriculture and Forestry University, Fuzhou, China

## Abstract

Chlorpyrifos-resistant (Rc) *Plutella xylostella* (DBM) shows higher wing-vein injury than chlorpyrifos-susceptible (Sm) DBM under heat stress in our previous study. To investigate the toxicological mechanisms of the differences in injury of wing vein between Rc- and Sm-DBM collected from Fuzhou, China, total ten cDNA sequences of wing-development-related genes were isolated and characterized in DBM, including seven open reading frame (ORF) (*ash*1, *ah2*, *ash3*, *ase*, *dpp*, *srf* and *dll* encoded 187 amino acids, 231 aa, 223aa, 397aa, 423aa, 229aa and 299aa, respectively), and three partly sequences (*salm*, *ser* and *wnt*-*1* encoded 614aa, 369aa and 388aa, respectively). The mRNA expression of the genes was inhibited in Rc- and Sm-DBM under heat stress, as compared with that an average temperature (25 °C). And, in general, significantly higher down-regulated expressions of the mRNA expression of the wing development-related genes were found in Rc-DBM as compared to those in Sm-DBM under heat stress. The results indicated that Sm-DBM displayed higher adaptability at high temperature because of significantly lower inhibition the mRNA expressions of wing-development-related genes. We suggest that significantly higher injury of wing vein showed in Rc-DBM under heat stress might be associated with the strong down-regulation of wing-development-related genes.

## Introduction

Insects are so numerous and widely distributed in nature because of their wings. The insect wing veins are not only an extraordinary skeleton to support wing, but also contains a catheter, a vascular gap and a nerve tissue^1^. The mechanism of insect wing-development pattern has mainly been focused in the biological model, *Drosophila melanogaster*. The formation of *Drosophila* wings consists of three cascade control pathways, i.e., anterior-posterior (A-P), dorsal-ventral (D-V), proximal-distal (P-D)^2^. The cascade control pathways determined the differentiation of the A-P, D-V and P-D in the wing disc by the expression of related genes, such as *engrailed* (en), *apterous* (ap), *arista-less* and other genes^3,4^. Thus the differentiation of the A-P is determined by the selective expression of *engrailed (en)* and the concentration gradient of *decapentaplegic* (*dpp*) protein^5–7^ in the wing disc. Development of D-V is determined by the selective expression of *AP* gene and the concentration gradient of the *wingless (wg)* protein^8,9^. And the base of the wing should develop into the distal wing lobe and the proximal middle thoracic dorsal plate, which is determined by the P-D^10,11^. As transcription factors, *decapentaplegic* (*dpp)*, *spalt-major-like (salm*), *serum response factor (srf)* and *wingless (wg)*, *serrste (ser) and achaete-scute complex (As-c)*, *Distal-less (dll)* were involved in the three pathway^8,12–14^.

The gene regulation pathway in the development of the wing veins is highly conserved among insects^15–17^. Both *wg* and *wnt* are the homeotic genes^18^, and *wnt* plays an essential role in signaling pathways in the insect^19–21^. *Wg* is a morphological element, and the expression of the target gene is regulated by its concentration^22–24^. If the *wg* is missing, wing leaf and hinge area cannot be formed^25^. *As-c* is a family gene and contains the domain of basic Helix-loop-helix (BHLH)^26^. The function of this domain is to regulate the development of peripheral nervous system^27^, the precursor form of external sensory organs^28^, wing scales and insect sensory bristles^29^ and achaete-scute complex^30^. In existing reports, the *Bombyx mori* contains 4 *AS-c* genes (*ash1*, *ash2*, *ash3*, *ase*)^31^. While Butterflies, Culicidae, *Apis mellifera*, and *Tribolium castaneum* also have *AS-c* genes. *Salm* is a kind of gene which encodes C2H2 zinc finger protein and unique functional domain Znf is contained in *salm*^32^. During the development of insect wings, it controls the formation of the wing veins^23^,the formation of the bronchial system^33^ and wing imaginal disc^24^. *Dpp* is a morphological element. If the *dpp* is missing, it causes the adult to produce small wings, and *salm* will not express^13^. *dll* plays a role in larval and adult appendage development. Shape and size of adult wings were determined by *dll*^16,34,35^. Also, it has a secondary role in the normal patterning of the wing margin^16,36^. *Ser* control expression of *cut* and *wg*^37,38^. And *srf* is required for the formation of inter-vein tissue of the wing^39,40^. The functions of wing-development-related genes, WG in *Sogatella furcifera*^41^ and AS-C in *Bombyx mori*^30^, were proved in the development of the wings based RNAi method. However, no literature can be found to confirm the function of wing-development-related genes in DBM, including using RNAi method.It was known that wing development of insects would be very important for insect’s biological and physiological traits and their behavior, such as migration^42^. The effect of temperature on the development of insect wings is also reported, wing sizes and shapes in *Drosophila* can be affected by temperatures^43^. Previous studies explored that temperature affects the size and shape of the wings^44–46^. Our previous studies provided firstly the proof that wing development of DBM affected by pesticides resistance, i.e.high-temperature experienced in pupal stage influenced the phenotype of wing venation in insecticide-resistant (Rc) and insecticide-susceptible (Sm) *P*. *xylostella*. While, Sm-DBM showed significantly higher thermal tolerance and lower damages of wing veins under heat stress than Rc-DBM^47^. The expression of the caspase-7 gene (as an essential marker of apoptosis) in Rc-DBM were significantly higher than that of Sm strains under heat stress, on the other hand, the induced responses of *hsp70s* (as an essential protected protein) in Sm-DBM were significantly higher than those in Rc-DBM^48–50^. Rc-DBM showed significantly lower fitness in hermotolerance, oxidative stress, apoptosis, heat-shock proteins and damages to reproductive cells, as compared to Sm-DBM^50^.

The facts suggest that higher heat tolerance and lower damages of veins in susceptible DBM might be associated with their significantly lower apoptosis gene-expression and higher hsp70 gene-expression under heat stress. However, the expression of wing-development-related-genes was unknown. Therefore, ten key genes in insect wing development pattern that control the development of wing and mRNA expression of the wing-development-genes of Rc- and Sm- *P*. *xylostella* under heat stress was studied in the present study.

## Results

### Cloning and sequencing analysis of wing development-related genes

**Figure 1:**
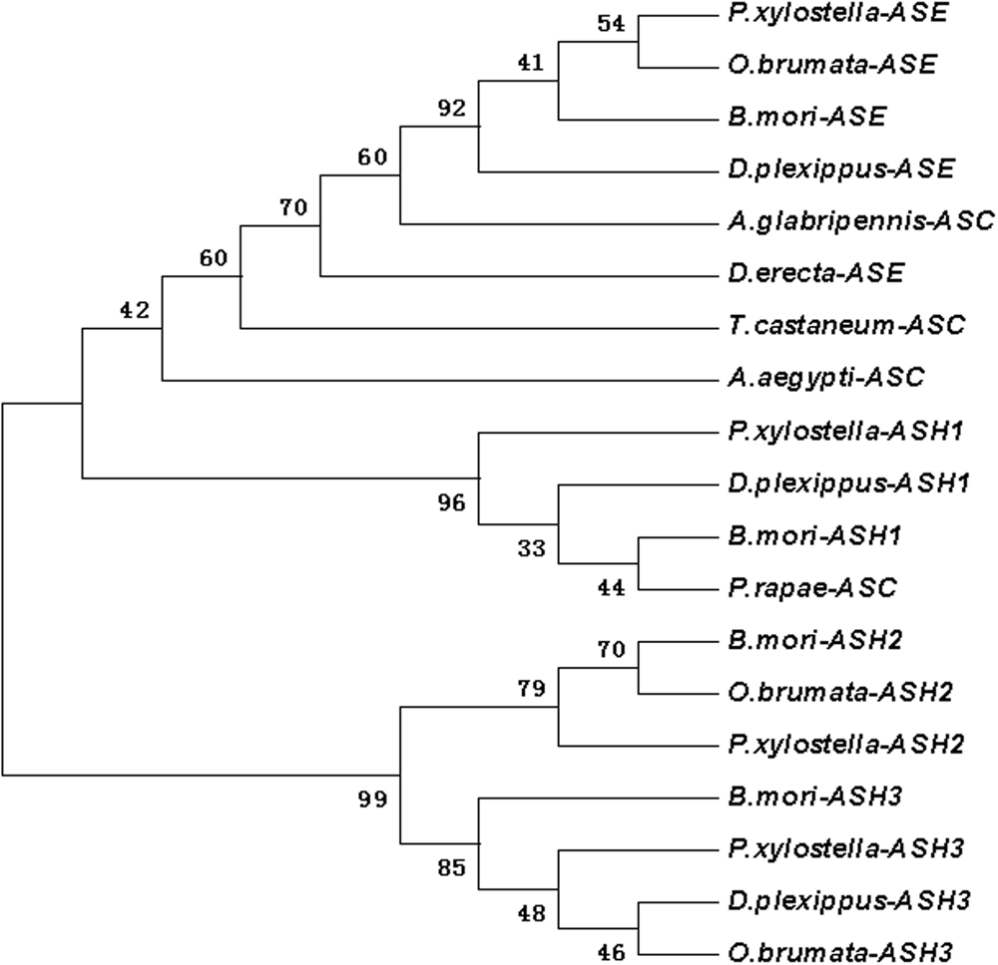
The phylogenetic analysis of achaete-scute homologue (ASH1, ASH2, ASH3, ASE) in DBM and other insects. *Plutella xylostella* ASE (AIZ67915.1); *Operophtera brumata* ASE (KOB69271.1); *Bombyx mori* ASE (NP_001098696.1); *Danaus plexippus* ASE (OWR43026.1); *Anoplophora glabripennis* ASC (XP_023310751.1); *Drosophila erecta* ASE (XP_001982390.1); *Tribolium castaneum* ASC (NP_001034537.1); *Aedes aegypti* ASC (XP_021712495.1); *Plutella xylostella* ASH1 (AIZ67914.1); *Danaus plexippus* ASH1 (OWR43025.1); *Bombyx mori* ASH1 (NP_001037416.1); *Pieris rapae* ASC (XP_022115666.1); *Bombyx mori* ASH2 (NP_001098692.1); *Operophtera brumata* ASH2 (KOB67667.1); *Plutella xylostella* ASH2 (AIZ67913.1); *Bombyx mori* ASH3 (NP_001098694.1); *Plutella xylostella* ASH3 (ALC76152.1); *Danaus plexippus* ASH3 (OWR43023.1); *Operophtera brumata* ASH3 (KOB69293.1).

**Figure 2:**
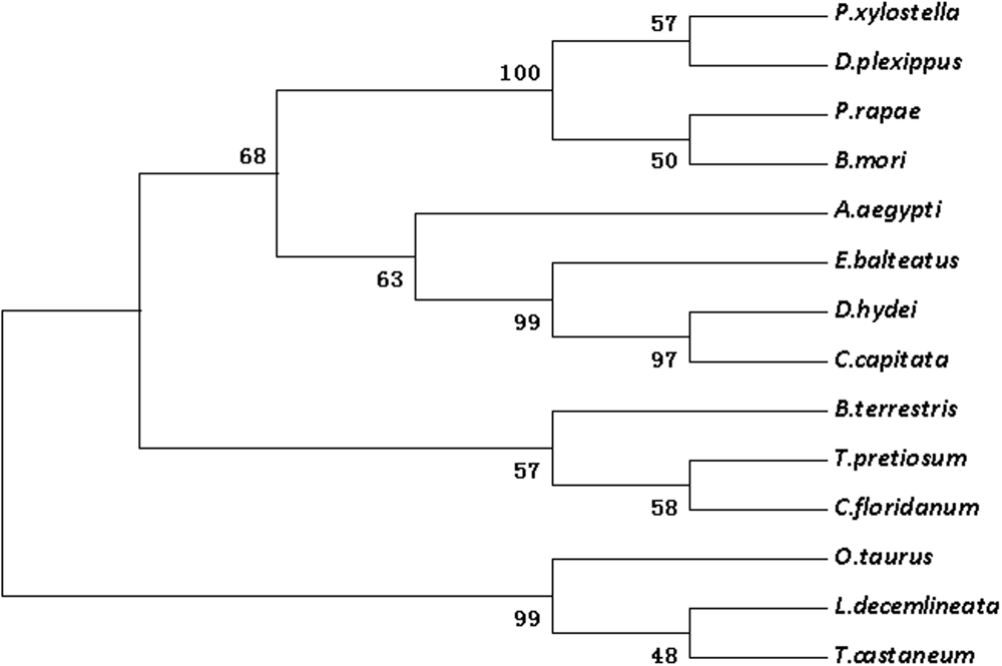
The phylogenetic analysis of Dpp in DBM and other insects. *Plutella xylostella* (ALC76150.2); *Pieris rapae* (XP_022119285.1); *Danaus plexippus* (OWR47416.1); *Bombyx mori* (XP_004929407.1); *Aedes aegypt*i (XP_021701909.1); *Trichogramma pretiosum* (XP_014230775.1); *Drosophila hydei* (XP_023170520.1); *Onthophagus taurus* (XP_022902496.1); *Episyrphus balteatus* (AEI25993.1); *Leptinotarsa decemlineata* (XP_023029182.1); *Copidosoma floridanum* (XP_014211661.1); *Ceratitis capitata* (XP_004530256.1); *Tribolium castaneum* (KYB26902.1); *Bombus terrestris* (XP_003394971.1).

**Figure 3:**
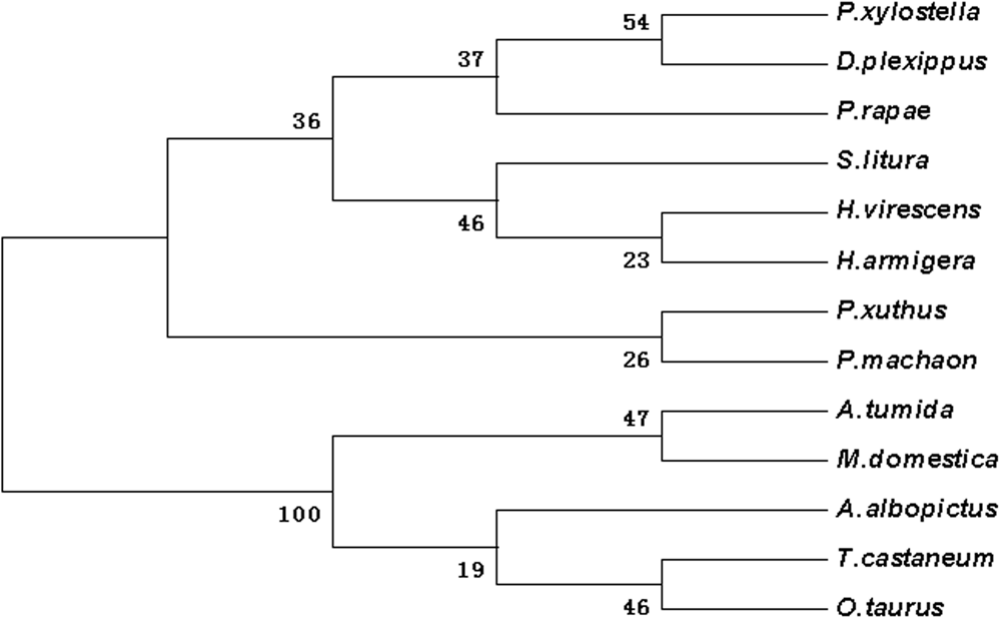
The phylogenetic analysis of SRF in DBM and other insects. *Plutella xylostella* (AIZ67912.1); *Papilio xuthus* (XP_013173375.1); *Onthophagus taurus* (XP 022907989.1); *Aethina tumida* (XP 019871043.1); *Musca domestica* (XP 011294945.1); *Aedes albopictus* (XP 019562715.1); *Pieris rapae* (XP 022115280.1); *Danaus plexippus* (OWR41122.1); *Papilio machaon* (XP 014357061.1); *Heliothis virescens* (PCG72703.1); *Helicoverpa armigera* (XP 021190114.1); *Spodoptera litura* (XP 022828228.1).

**Figure 4:**
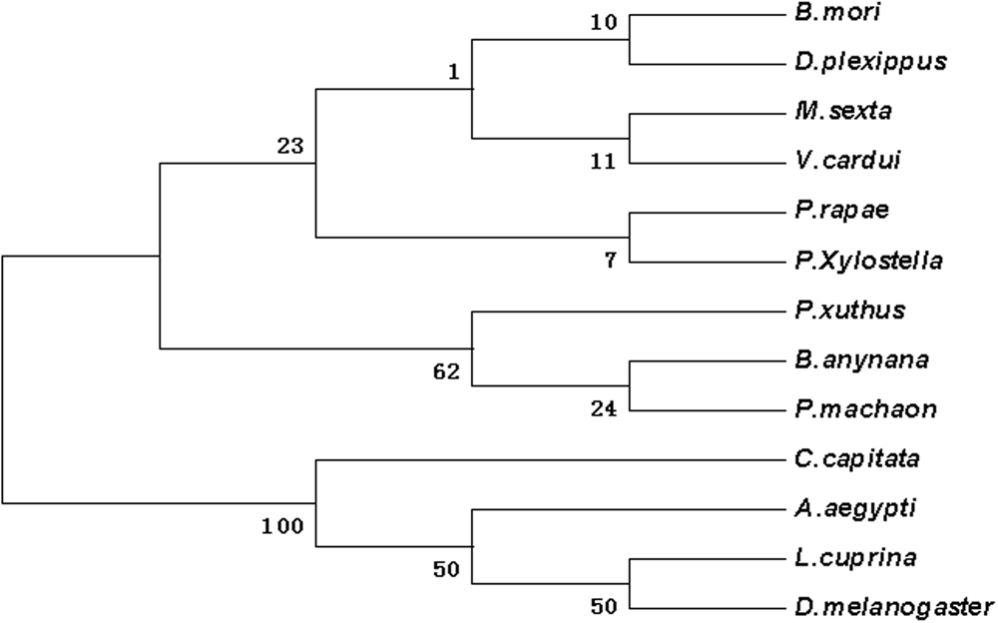
The phylogenetic analysis of dll in DBM and other insects. *Bicyclus anynana* (AAL69325.1); *Pieris rapae* (XP_022120979.1); *Bombyx mori* (XP_012551909.1); *Manduca sexta* (AAT39558.1); *Vanessa cardui* (AJS19035.1); *Papilio machaon* (KPJ10995.1); *Danaus plexippus* (OWR43046.1); *Papilio xuthus* (KPI96576.1); *Aedes aegypti* (XP_021698712.1); *Lucilia cuprina* (KNC21963.1); *Drosophila melanogaster* (NP_726486.1); *Ceratitis capitata* (XP_012161414.1).

**Figure 5:**
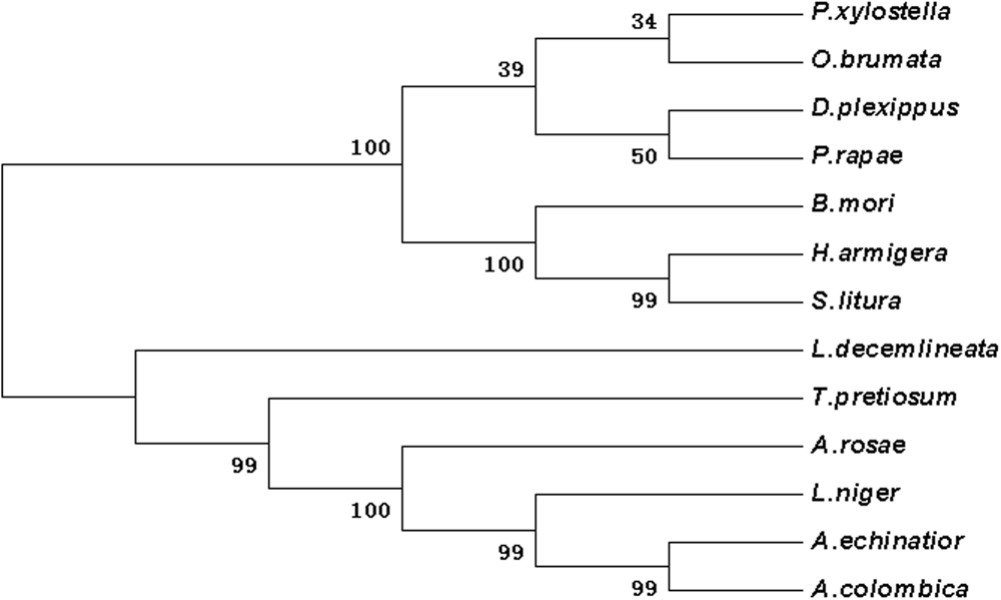
The phylogenetic analysis of salm in DBM and other insects. *Plutella xylostella* (ALC76153.2); *Danaus plexippus* (OWR44146.1); *Operophtera brumata* (KOB70391.1); *Bombyx mori* (XP_012549950.1); *Pieris rapae* (XP_022112446.1); *Helicoverpa armigera* (XP_021184031.1); *Spodoptera litura* (XP_022820679.1); *Leptinotarsa decemlineata* (XP_023026015.1); *Lasius niger* (KMR01384.1); *Trichogramma pretiosum* (XP_023316968.1); *Athalia rosae* (XP_020709204.1); *Acromyrmex echinatior* (EGI68679.1); *Atta colombica* (KYM93127.1).

**Figure 6:**
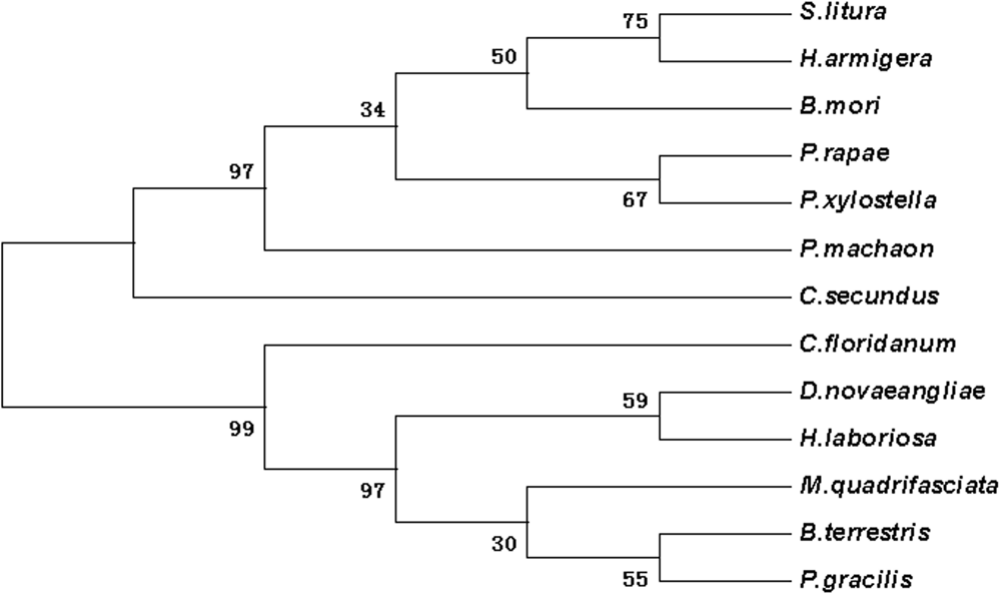
The phylogenetic analysis of ser in DBM and other insects. *Spodoptera litura* (XP_022821559.1); *Pieris rapae* (XP_022130556.1); *Helicoverpa armigera* (XP_021188794.1); *Bombyx mori* (XP_012549285.1); *Papilio machaon* (KPJ16753.1); *Cryptotermes secundus* (PNF27227.1); *Dufourea novaeangliae* (KZC14437.1); *Bombus terrestris* (XP_003399363.1); *Habropoda laboriosa* (KOC62545.1); *Pseudomyrmex gracilis* (XP_020288292.1); *Melipona quadrifasciata* (KOX79304.1); *Copidosoma floridanum* (XP_014211886.1).

**Figure 7:**
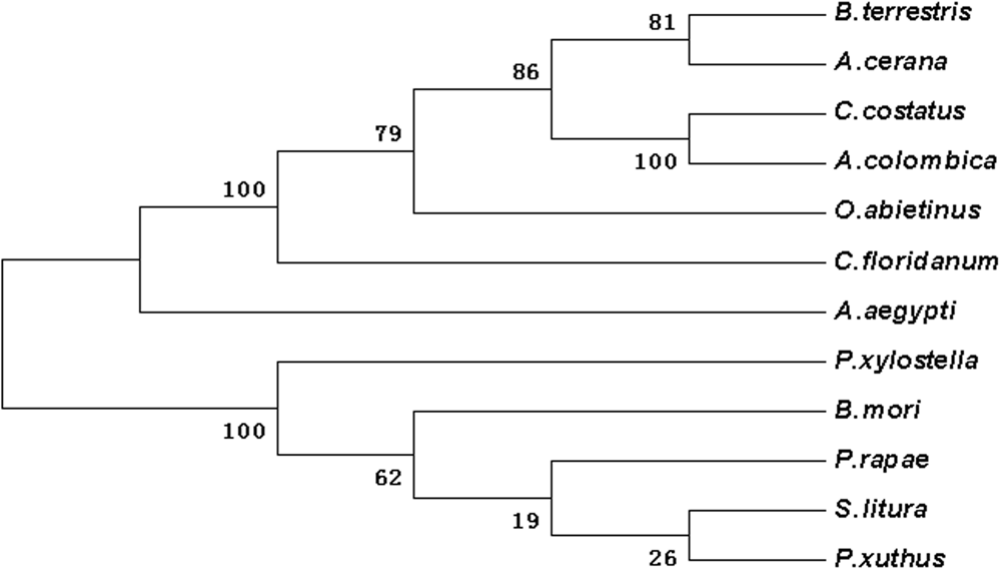
The phylogenetic analysis of wnt-1 in DBM and other insects. *Plutella xylostella* (ALC76151.1); *Spodoptera litura* (XP_022820536.1); *Pieris rapae* (XP_022116444.1); *Bombyx mori* (NP_001037315.1); *Papilio xuthus* (KPI94016.1); *Aedes aegypti* (XP_021702999.1); *Copidosoma floridanum* (XP_014212755.1); *Orussus abietinus* (XP_012289230.1); *Bombus terrestris* (XP_003393164.1); *Apis cerana* (PBC26820.1); *Cyphomyrmex costatus* (KYN01512.1); *Atta colombica* (KYM88337.1).

Based on the initial fragment sequence, the 3′- and 5′-end fragment were obtained by 3′- and 5′-RACE, respectively. Six full-length cDNA sequences (873; 809; 1012; 1799; 1891 and 949 bp for *ash1*; *ash2*; *ash3*; *ase*; *dpp* and *srf*, respectively) and four partial cDNA sequences (1541; 1885; 1196 and 1213 bp for *dll*; *salm*; *ser* and *wntl*, respectively) of wing development-related genes from DBM were gained by editing and splicing the cloned sequence (Table 1). The ORF sequence was 564 bp of *ash1* (67–630 bp), 696 bp of *ash2* (62–757 bp), 672 bp of *ash3* (117–788 bp), 1194 bp of *ase* (144–1337 bp), 1272 bp of *dpp* (342–1613), 690 bp of *srf* (20–709 bp). The open reading frame (ORF) of the *ash1*, *ah2*, *ash3*, *ase* encodes 187, 231, 223 and 397 amino acids, respectively, and analysis showed that all of them contain HLH domain (55–116, 77–140, 68–131 and 98–162 aa, respectively). The ORF of *dpp* encodes 423aa that contain DWA and DWB domain (39–148aa and 227–399aa, respectively). The ORF of *srf* encodes 229aa that contain MADS domain (56–115aa). The four partial cDNA sequences (*dll*, *salm*, *ser* and *wntl*) encodes 299, 614, 369 and 388 aa, respectively (Table 2). Functional domain analysis of these amino acid sequences (*dll*, *salm*, *ser*, *wntl*), the HOX (129–191 aa), ZnF_C2H2 (86–108, 114–136, 431–453, 459–481 and 491–513 aa), Coiled-coil (105–135 aa) and *wntl* (56–388 aa) domain were found respectively (Tables 1 and 2, Figs 1–7 and S1–S10).

**Table 1:** Analysis of nucleotide and inferred amino acid sequences of s cDNA of DBM.

	Ash1	Ash2	Ash3	Ase	Dpp	Srf
GenBank acc. no.	KJ158244	KJ158243	KP245732	KJ466144	KP245730	KJ158242
Full-length (bp)	873	809	1012	1799	1891	949
ORF	67–630	62–757	117–788	144–1337	342–1613	20–709
Putative protein (aa)	187	231	223	397	423	229
Domain	HLH	HLH	HLH	HLH	DWA DWB	MADS
	55–116	77–140	68–131	98–162	39–148 227–399	56–115

**Table 2:** Analysis of nucleotide and inferred amino acid sequences of s cDNA of DBM (partial cDNA).

	dll	salm	ser	wnt
Length (bp)	1541	1885	1196	1213
ORF	156–1055	42-	90-	50-
Putative protein (aa)	299	614	369	388
Domain	HOX 129–191	ZnF_C2H2 86–108 114–136 431–453 459–481 491–513	Coiled coil 105–135	WNT1 56–388

According to the Blast results, the higher homogeneities of amino acids in the wing development-related genes with other insects were as follows. *ash1* had presented 78, 79, 79 and 81 percent with *Bombyx mori* (NP_001037416), *Danaus plexippus* (OWR43025), *Helicoverpa armigera* (XP_021183890) and *Spodoptera litura* (XP_022829400.1), respectively (Fig. S1). *ash2* had exhibited 85, 84 and 83 percent with *B*. *mori* (NP_001098692), *S*. *litura* (XP_022829477), and *H*. *armigera* (XP_021183904), respectively (Fig. S2). *ash3* had shown 68, 62 and 61 percent with *B*. *mori* (NP_001098694), *D*. *plexippus* (OWR43023) and *Operophtera brumata* (KOB69293), respectively (Fig. S3). *ase* had depicted 72, 70, 68 and 66 percent with *Pieris rapae* (XP_022115661), *H*. *armigera* (XP_021183923), *D*. *plexippus* (OWR43026) and *B*. *mori* (NP_001098696), respectively (Fig. S4). *dpp* had illustrated 98, 98, 82 and 81 percent with *B*. *mori* (XP_004929407), *D*. *plexippus* (OWR47416), *Drosophila melanogaster* (NP_001259992) and *Lasius niger* (KMQ88665), respectively (Fig. S5). Similarly, *srf* had demonstrated 92, 92, 91, 90 and 68 percent with *H*. *armigera* (XP_021190114), *S*. *litura* (XP_022828228), *P*. *rapae* (XP_022115280), *B*. *mori* (XP_012552250) and *Tribolium castaneum* (NP_001139383), respectively (Fig. S6). Likewise, *dll* had shown 95, 94, 93, 90 and 60 percent with *Manduca sexta* (AAT39558), *Vanessa cardui* (AJS19035), *P*. *rapae* (XP_022120979), *B*. *mori* (XP_012551909) and *D*. *melanogaster* (NP_726486), respectively (Fig. S7). *salm* had given 83, 82, 82, 69 and 67 percent with *O*. *brumata* (KOB70391), *D*. *plexippus* (OWR44146), *P*. *rapae* (XP_022112446), *B*. *mori* (XP_012549950) and *H*. *armigera* (XP_021184031), respectively (Fig. S8). *ser* had shown 74, 73, 73, 73, 72 and 38 percent with *H*. *armigera* (XP_021188794), *P*. *rapae* (XP_022130556), *S*. *litura* (XP_022821559), *Papilio machaon* (KPJ16753), *B*. *mori* (XP_012549285) and *Cryptotermes secundus* (PNF27227), respectively (Fig. S9). *wnt-1* had presented 88, 87, 82 and 81 percent with *P*. *rapae* (XP_022116444), *S*. *litura* (XP_022820536), *Papilio xuthus* (KPI94016) and *B*. *mori* (NP_001037315), respectively (Fig. S10). Phylogenetic analysis revealed that wing-development-related genes from *P*. *xylostella* were clustered together with other Lepidoptera insects (Figs 1–7).

### Expressions of wing-development-related genes under heat stress in pupae

**Figure 8:**
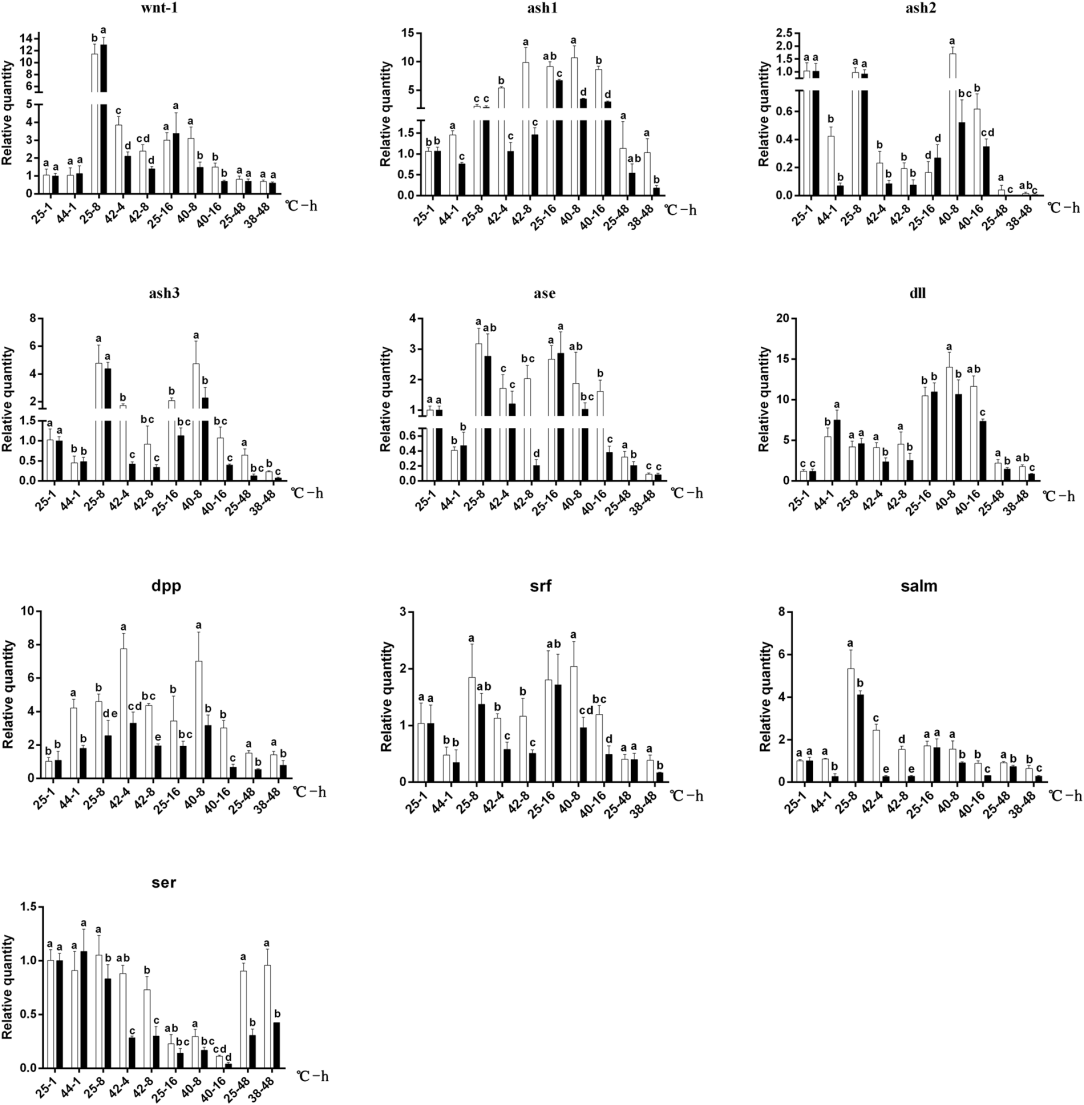
Effects of heat stress on the expression of wnt-1, ash1, ash2, ash3, ase, dll and dpp, srf, salm ser in Rc (black) and Sm (white) pupa DBM. Abscissa: temperature (°C)-treated time (h). Ordinate: relative quantity expression of the genes. Pupae newly formed at 25 °C from both Rc and Sm populations were incubated by four temperature treatments, that is, 25 or 44 °C for 1 h (25–1 or 44–1), 25 or 40 °C for 8 (25–8 or 40–8) or 16 h (40–16), 25 or 42 °C for 4 (25–4 or 42–4) or 8 h (42–8), and 25 or 38 °C for 48 h (25–48 or 38–48), respectively. Living pupae after heat stress were allowed to recovery for 1 h at 25 °C before they were used for extracting mRNA. The values in each group of the four temperature treatments were used for statistical analysis, respectively, in Rc and Sm DBM. Lower-case letter indicates significant difference in mRNA expression in each temperature treatment group (Duncan test, P ≤ 0.05).

In general, as compared to 25 °C, the mRNA expression levels of *ash2*, *ash3*, *ase*, *srf* were significantly down-regulated both in Sm- and Rc-pupae under heat stress, and non-significant differences were observed between Sm- and Rc-strain, whereas significantly down-regulated at 42 °C for 4 or 8 h in *ash3*, at 42 °C for 8 h and 40 °C for 10 h in *ase* and at 42 °C for 4 and 8 h and 40 °C for 8 and 16 h in *srf* were found in Rc- strain, as compared to those in Sm-strain (Fig. 8). Although Sm- and Rc-pupae displayed the same level of mRNA expression of *wnt-1* in all groups, heat stress resulted in significantly down-regulated expression of *wnt-1* in Rc- pupae (Fig. 8). Sm- and Rc-pupae showed similar level of mRNA expression of ash1 at 25 °C in all group, however, significant higher down-regulated expressions of ash1 were found in Rc-pupae at 42 °C for 4 or 8 h, 40 °C for 8 or 16 h and 38 °C for 48 h (Fig. 8). Rc-pupae showed significantly down-regulation mRNA expression of *dpp* and *salm* in almost all of the heat stress groups (Fig. 8). Rc-pupae displayed the significantly lower expressions of *dll* and *ser* at 42 °C for 4 or 8 h, 40 °C for 8 or 16 h and 38 °C for 48 h (Fig. 8). In general, Rc-pupae displayed significantly lower expression of mRNA than Sm-pupae under heat stress for the wing-development-related genes under heat stress in the the groups, and no significant differences in basal (at 25 °C) expression of most genes between Sm- and Rc-pupae (Fig. 8).

There was a distinct feature in the group of (25 °C-16h, 40 °C for 8 h and 16 h) that most genes (*ase*, *dll*, *srf*, *dpp*) were well expressed in Sm-strain under heat stress, especially at 40 °C (for 8 h or 16 h). Rc- pupae displayed significantly lower expression of mRNA than Sm-pupae under heat stress at 40 °C (Fig. 8).

In the group of (25 °C-48h; 38 °C-48h), all wing-development-related genes showed lower mRNA expressions level than other groups, except *ser*. And most of them showed the same pattern that is Rc-pupae showed significantly lower expression of mRNA than Sm-pupae under heat stress, but interestingly, the same pattern also happens in 25 °C, that is different from other groups (Fig. 8).

## Discussion

In the present study, ten cDNA sequences of wing-development-related genes were identified from the diamondback moth, *P*. *xylostella*. The results of amino acid sequence alignment showed that the *ash1*, *ash2*, *ash3*, *ase*, *dpp*, *srf*, *dll*, *salm*, *ser* and *wnt-1* protein of *P*. *xylostella* was similar to the homologous protein in other species. Because DBM belongs to Lepidoptera, the amino acid sequence of *P*. *xylostella* was compared with that of another Lepidopteran insect, *B*. *mori*. Analysis of amino acid sequences homogeneity between *P*. *xylostella* and *B*. *mori* had shown 66–98 percent similarity, higher conservation in the structural domain. In addition, the phylogenetic analysis revealed that wing-development-related genes from *P*. *xylostella* were clustered together with other Lepidopteran insects. The above analysis confirms that the obtained gene sequences are our target genes.

In general, based on the selected Rc- and Sm-DBM pupae, significant inhibitions of mRNA expression (down-regulation of wing-development-related genes) were found in all of the wing-development-related genes in both Sm and/or Rc-DBM pupae under heat stress including at 40 °C, 42 °C, 44 °C and/or 38 °C treatments, although there were several exceptions. Although no significant differences in basal expression (at 25 °C) of most genes between Sm- and Rc-pupae, significantly higher inhibitions on the mRNA expression of the genes were found in Rc-DBM in many cases.

The fitness cost in resistant insect species is a general tendency and found in many insect species^51^. Insecticide-resistant insects showed significant fitness cost in life-history, behavior and physiological traits^48^. However, the researches were carried out under a suitable temperature^48^. In our previous study, it was confirmed that insecticide-resistant DBM showed significant fitness cost under heat stress. Rc-DBM showed significantly lower fitness under heat stress in hermotolerance, fecundity, oxidative stress, apoptosis, heat-shock proteins and damages to reproductive cells, as compared to Sm-DBM^47–50^. In addition, higher wing-vein injury under heat stress in Rc-DBM was found as compared to that in Sm-DBM^47^. It was the first evidence of morphogenesis as the fitness cost caused by insecticide resistance in insects. However, the mechanisms should be investigated.

The present study was aimed to study the effects of high temperature on the mRNA expression of the wing development-related genes in insecticide-resistant and –susceptible DBM. Rc- or Sm-DBM pupae strains were reared and collected for temperature treatments at the same time. The results obtained in present study indicated that heat stress would resulted in significantly higher inhibitions on the mRNA expressions of wing development-related genes in Rc-DBM pupae strains, and significant fitness costs were existed in Rc-DBM pupae trains. Significantly higher inhibitions on mRNA expression of the wing development-related genes under heat stress in Rc-DBM was confirm firstly. The results indicated that higher damage in development of wing veins in Rc-DBM under heat stress^47^ might be associated with significantly higher inhibitions on the mRNA expressions of wing development-related genes. The fitness cost in resistant DBM in our previous studies^47–50^ and in the present study might be linked partly to the resistant alleles ace1R – the allele conferring the resistance to several to several OP insecticides^48^. Although the mRNA expression of the genes was studied by qPCR in present study, the RNAi of the genes should be conducted to study the functions of the genes in our future study.

The wing development of insects would be very important for insect’s evolution and adaptability to environment^30^. It would be important that when designing insect management program, fitness cost caused by insecticide resistance should be considered to maximize the effect of insecticides and minimize costs and residues of controlling insects.

## Methods

### Sources of insects

Resistant and Sensitive strains of DBM were long-term reared in our laboratory. The Sm-strain is the most sensitive to chlorpyrifos whereas Rc-strain is highly resistant to this insecticide. These strains were gained from a population collected from Shangjie (34°480 N, 113°180E) (Fuzhou, Fujian, China). Details of the selection of these two strains are provided elsewhere^50^. No specific permissions were required for our collection of *P*. *xylostella*, because the scientists were welcome to collect the insect sample from the farmer’s crucifer fields in order to control the pest insects. The field studies did not involve endangered or protected species.

### Cloning wing development-related genes

Fourth instar larvae of Sm-DBM were selected and reared at 25 °C temperature. Then on the third day, F1 progenies were used for the total RNA extraction.

#### Amplification of the initial fragments of wing development-related genes

Total RNAs were extracted according to the manufacturer’s instructions for the MiniBEST Universal RNA Extraction Kit (TaKaRa Bio Inc.). First-strand cDNAs were synthesized from 5 μg of total RNAs using PrimeScript™ II 1^st^ Strand cDNA Synthesis Kit (TaKaRa Bio Inc.). For amplification of the initial fragments of wing development-related genes, specific primers were designed (Table 3). PCR conditions were as follows; 94 °C denaturation for 3 min, followed by 40 cycles of 94 °C for 30 s, an annealing step at 52 °C (52 °C for *dll*; 54 °C for *ash1*, *ash3*, *ase*, *ser*; 55 °C for *ash2*; 56.5 °C for *salm*; 57 °C for *srf*; 59.5 °C for *dpp*; 61 °C for *wnt-l*) for 1–2 min, an extension step at 72 °C for 2–3 min, and a final extension step at 72 °C for 7–10 min.

**Table 3:** Sequences of primers used for cloning wing development-related genes cDNAs of DBM.

Names of primers	Sequences of Primers (5′–3′)	Product size(bp)	Names of primers	Sequences of Primers (5′–3′)	Product size(bp)
For initial fragment(s)			For RACE		
Ash1-F	5′–GGAGGAACGCCAGGGAG–3′		3′Ash1-F1	5′–CGGTTGTTCAGAGTCGCA–3′	
Ash1-R	5′–GTTGTTGCCACCACGATA–3′	401	3′Ash1-F2	5′–CCCACCTGTCGAGACACCTA–3′	336
Ash2-F	5′–ACTACAACTCGCCCAAGGTCA–3′		5′Ash1-R1	5′–CAGGGCTCATTGGTTCATAGG–3′	
Ash2-R	5′–CGGAATAGGCGGAGGACA–3′	593	5′Ash1-R2	5′–TGACACAGCCGAGGGGATG–3′	309
Ash3-F	5′–GGGAGAGGAACCGAGTG–3′		3′Ash2-F1	5′–GAGAGGAACCGAGTGAAGCAAGT–3′	
Ash3-R	5′–GTACTCCACCACCATGCGG–3′	155	3′Ash2-F2	5′–GGAGAACATCCCCAACGGC–3′	287
Ase-F	5′–ACATACCTGAGGAAGTCGC–3′		5′Ash2-R1	5′–CTGCCGTAGAATAACCCGTC–3′	
Ase-R	5′–CTTCTCCTGCCACCATTT–3′	800	5′Ash2-R2	5′–ACTTGCTTCACTCGGTTCCTC–3′	321
Dll-F	5′–GAATACAAACCCCCGAC–3′		3′Ash3-F1	5′–GCCTTACCAAACCCTACCTG–3′	
Dll-R	5′–ATGGTCCTGGGCTTCCTC–3′	507	3′Ash3-F2	5′–ACCGAGTGAAGCAAGTGAACGACG–3′	679
DPP-F	5′–GGGATGGAAGCAGGGTGATG–3′		5′Ash3-R1	5′–GTACTCCACCACCATGCGG–3′	
DPP-R	5′–GTCCTTTGGAACGGGAGCAG–3′	418	5′Ash3-R2	5′–ATGCGGAGCGTGTCCACCTTGC–3′	466
Salm-F	5′–GCGGATGAACTGGCATTTAG–3′		3′Ase-F	5′–TACACGTGCAGATGAACCACAACT–3′	725
Salm-R	5′–CGCCGACGGTTTACTGAGAT–3′	1414	5′Ase-R1	5′–TGGACCACCGTCTTCCTCTT–3′	
Ser-F	5′–CGGTCAGGTCCCGATTA–3′		5′Ase-R2	5′–GTATTCGACAGCCATGCGTAGT–3′	599
Ser-R	5′–AGGAACCCACCGTAGCG–3′	1196	3′Dpp-F1	5′–TCAACCCGTACCACTACAAGCG–3′	
SRF-F	5′–CCCACCATCCAACGGCAA –3′		3′Dpp-F2	5′–AACCCGTACCACTACAAGCGA–3′	564
SRF-R	5′– GTCGGGCGAGTTGAGGCA–3′	274	5′Dpp-R1	5′–TCCAGTTCCTCAATAGCCCC–3′	
Wnt-l-F	5′–CCGACGCTGGAACTGCTC–3′		5′Dpp-R2	5′–CTTCACCAGACTGTCCACCG–3′	1155
Wnt-l-R	5′–ACCTTCTCCGTGTGGCAG–3′	880	3′SRF-F1	5′–CAAGCGGAAGACAGGGATT–3′	
For ORF			3′SRF-F2	5′–GCAACACGCAAACTCCAGC–3′	594
Ash1-ORF-F	5′-GACTCTACACTCGCGATG-3′		5′SRF-R1	5′–GCTGGAGTTTGCGTCTTGC–3′	
Ash1-ORF-R	5′-CAAAGCTCCGATACATGATAC-3′	712	5′SRF-R2	5′–AATCCCTGTCTTCCGCTTG–3′	268
Ash2-ORF-F	5′-GCGACCGCCACTCACCC-3′		5′Wnt-l-R1	5′–AGCCGCCCCACTTCCACACGC–3′	
Ash2-ORF-R	5′-TGTTATTTCTGTTGCCACCACG-3′	721	5′Wnt-l-R2	5′–CGCTCGTGATGGCGTAGATG–3′	443
Ash3-ORF-F	5′-AACCCTACCTGTTTACGA-3′				
Ash3-ORF-R	5′-CAATGTTTACACAATGCCA-3′	860	UPM*(long)	5′-CTAATACGACTCACTATAGGGCAAGCAGTGGTAACAACGC	
Ase-ORF-F	5′-GCGACTAAACCAAAACAAACACC-3′			AGAGT-3′	
Ase-ORF-R	5′-CGTAACTTAGAGGACAAACC-3′	1513	UPM*(short)	5′-AAGCAGTGGTAACAACGCAGAGT-3′	
Dpp-ORF-F	5′-CGATGGTGGATTAGGCA-3′		NUP*	5′-AAGCAGTGGTAACAACGCAGAGT-3′	
Dpp-ORF-R	5′-TGCGGCTAAGATACTGAGGAG-3′	1374			
SRF-ORF-F	5′-GACCAGTGCTACGACGAG−3′				
SRF-ORF-R	5′-GTCCCGAACCGAAAACCT-3′	773			

* The primer sequences were as described in the SMARTer® RACE 5′/3′ Kit.

#### Rapid Amplification of cDNA Ends (RACE) of wing development-related genes

For 3′- and 5′-RACE, the first-strand cDNAs were separately constructed from 1 μg of total RNA according to the SMARTer^®^ RACE 5′/3′ Kit (Clontech Laboratories, Inc.). According to the cloned intermediate target gene fragment and the nested PCR principle, 3′- and 5′-RACE specific primers were designed by using Primer 5.0 software, respectively (Table 3). PCR Program was set as follows; 94 °C for 3 min; 94 °C 30 sec, 68 °C 30 sec, 72 °C 3 min for 25 cycles, and 72 °C for 7 min.

#### Amplification of ORFs

To identify the edited full-length sequences, ORFs were amplified by using forward and reverse primers corresponding to the 5′ and 3′-ends of the full-length sequences, respectively (Table 3). PCR conditions were 94 °C for 5 min, followed by 35 cycles of 94 °C for 30 sec, 52 °C (52 °C for *ash1*, 55 °C for *ash2*, 47 °C for *ash3*, 52 °C for *ase*, 55 °C for *dpp*, 57 °C for *srf*) for 30 sec, and 72 °C for 1–2 min, finally 72 °C for 7–10 min. The initial fragments, 3′- and 5′-RACE fragments and the ORF fragments were cloned and sequenced by Shanghai Biosune Biotechnology Co., Ltd., Shanghai, China.

### Real-time quantitative PCR (qPCR)

#### Temperature shock

According to the previous study of Zhang *et al*.^47^, the pupae were grouped into four groups and treated with heat stress at four different temperatures, i.e., 38 °C for 48 h; 40 °C for 8 h and 16 h; 42 °C for 4 h and 8 h, and 44 °C for 1 h, whereas the control group was maintained at 25 °C in the four groups, respectively. After heat stress, the pupae were allowed to recover for 1 h at 25 °C, before being used for detecting mRNA expression.

#### Determination of mRNA expression

After RNA extraction, cDNAs were synthesized from 0.5 μg of total RNAs using PrimeScriptTMRT reagent Kit (TaKaRa Bio Inc.). The primers used for qPCR of these genes are listed in Table 4, while the primers for *β*-actin (house-keeping gene) were used as the endogenous control. qPCR was executed using an Applied Biosystems 7500 Real-Time PCR System (AB, Life Technologies) with SYBR Premix Ex Taq™ (TaKaRa Bio Inc.), and conditions were set as following: 95 °C for 30 sec; 40 cycles of 95 °C for 5 sec, 60 °C for 34 sec; Melt Curve: 95 °C for 15 sec, 60 °C for 1 min, and 95 °C for 15 sec. The homogeneity of the PCR products was confirmed by melting curve analysis. The expression level of each gene was calculated according to the threshold cycle (CT) and the relative expression was calculated using the Livak method (2^(−ΔΔCt)^)^52^. Thus, the normalized expression value of the target gene was calculated by comparing the expression value of the target gene with *β*-actin^49^. All data obtained from qPCR were analyzed using the Statistical Product and Service Solutions (SPSS). Three biological replications, with ten insect individuals for each replication, were used in the expression of mRNA.

**Table 4:** Sequences of primers used for qPCR of wing development-related genes of DBM.

Primers	Sequences of primers (5′-3′)	Gene names	Product size
β-actin-F	5′-ACCGGTATCGTGCTGGACTC-3′	β-actin	239
β-actin-R	5′-GCCATCTCCTGCTCGAAGTC-3′		
Pxw1-F	5′-GGTGAGGACGGTTGTTCAGAGT-3′	Ash1	171
Pxw1-R	5′-CTTCAGGGCTCATTGGTTCATA-3′		
Pxw2-F	5′-ACTACAACTCGCCCAAGCTTA-3′	Ash2	248
Pxw2-R	5′-ACTTGCTTCACTCGGTTCCTC-3′		
Pxw3-F	5′-GCCTTACCAAACCCTACCTG-3′	Ash3	167
Pxw3-R	5′-CGCTATCGGCACCATTTT-3′		
Pxw4-F	5′-CCATCATCAGACCTCCTAGC-3′	Ase	126
Pxw4-R	5′-TGTCGGATTCACCACAAAAG-3′		
Pxw5-F	5′-CAAGAACCGACGCTGGAAC-3′	Wnt	115
Pxw5-R	5′-CGCTCGTGATGGCGTAGAT-3′		
Pxw6-F	5′-GGGGTATCCCTTCCCTCCTAT-3′	dll	152
Pxw6-R	5′-ACCGCATTTCTCATCCTTCG-3′		
Pxw7-F	5′-TACAGGTATCCCACCGCAAGG-3′	dpp	166
Pxw7-R	5′-TCCTTTGGAACGGGAGCAG-3′		
Pxw8-F	5′-TGGACACCGTCGTCATCAAG-3′	ser	141
Pxw8-R	5′-CCTTCACGGCATCCACATCT-3′		
Pxw9-F	5′-CAATGAGCCCTTCTTCAAACACC-3′	salm	222
Pxw9-R	5′-TGTTCTGGTATTGGATTAGGGTTCA-3′		
Pxw10-F	5′-ACCATCCAACGGCAAGAAGA-3′	srf	168
Pxw10-R	5′-CGACGCGACTAGCAACATCA-3′		

#### Phylogenetic tree analyses

The phylogenetic analysis of achaete-scute homologue in DBM and other insect was carried out by using the maximum likelihood method (MEGA 6.0).

### Statistical analysis

The data of mRNA expression were analyzed by probit analysis^53^ using a DPS data processing system^54^.

## Electronic supplementary material

Supplemental information
