# Supplementary material for: Cloning of Wing-Development-Related Genes and mRNA Expression Under Heat Stress in Chlorpyrifos-Resistant and -Susceptible Plutella xylostella
Source: Sci Rep. 2018 Oct 15;8:15279. doi: 10.1038/s41598-018-33315-z (PMC6189056; doi:10.1038/s41598-018-33315-z)
Supplement: Supplementary file 1 — Supplemental information [file 41598_2018_33315_MOESM1_ESM.pdf]

Supplemental information

Cloning of wing-development-related genes and mRNA expression under heat stress in chlorpyrifos-resistant and -susceptible *Plutella xylostella*

Xue Zhun Chen[1], Qi Xing Hu[1], Qi Qing Liu[1], Gang Wu[1,*]

```
P.xylostella     MPMAAIHSYQSLGHNQ.....QN.AQRRLAPAPMDPSRCKKKSSYLHQQYPAQPASVARR    54
Bombyx.mori      MPMAAIHGYQNLGVDQKLTHLQTPTPRRLAPAPVDTTRCKKRS.YLHQPYPTQPASVARR    59
D.plexippus      MPMAAVHAYQNLGVNQKLTQLQN..ARRLAPAPVD.ARCKKRS.YLHQTYPTQPASVARR    56
H.armigera       MPMAAIHTYQNLGVDQKLTHLQSQAPQRLAPAPVDPNRCKKKS.YLHQPYPTQPASVARR    59
S.litura         MPMAAIHTYQNLGVDQKLTHLQSQAPRRLAPAPVDPGRCKKKS.YLHQPYPTQPASVARR    59
Consensus        mpmaa h yq lg  q     q     rlapap d  rckk s ylhq yp qpasvarr

P.xylostella     NARERNRVKQVNNGFAALRQHIPSAVSAAIIGGRGSSRKLSKVDTLKLAVEYIRSLKRLL   114
Bombyx.mori      NARERNRVKQVNNGFAALRQHIPSAVTAALAGGRGSSRKLSKVDTLRLAVEYIKSLKRLL   119
D.plexippus      NARERNRVKQVNNGFAALRQHIPSAVTAALAGGRGSSRKLSKVDTLRLAVEYIRSLKRLL   116
H.armigera       NARERNRVKQVNNGFAALRQHIPSAVSAALAGGRGSSRKLSKVDTLRLAVEYIKSLKRLL   119
S.litura         NARERNRVKQVNNGFAALRQHIPSAVSAALAGGRGSSRKLSKVDTLRLAVEYIKSLKRLL   119
Consensus        narernrvkqvnngfaalrqhipsav aa  ggrgssrklskvdtl laveyi slkrll

P.xylostella     EEGEDGCSESQTSLGLG.SGAQTPPSSDASHSPAPSFVSESSAGPTCRDTYDSYEPMSPE   173
Bombyx.mori      DESDDGCSDTQLGLGLSTTGSQTPPSSEESHSPAPSFVSESSAGPNCHDVYDAYEPMSPE   179
D.plexippus      EESEEGCNEAQMSLGLSSNGPQTPPFSEESHSPAPSLHSEGSTGPNCQDAYDSYEPMSPE   176
H.armigera       EESDEGCSDTQSGIVLTSSGPHTPPLSEESHSPAPSFVSESSAGPSCHDAYDSYEPMSPE   179
S.litura         EESEEGCSESQSGLVLSNSGQHTPPLSEESHSPAPSFVSESSVGPSCHDAYDSYEPMSPE   179
Consensus         e   gc   q    l   g  tpp s  shspaps  se s gp c d yd yepmspe

P.xylostella     DEELLDVISWWQQ                                                  186
Bombyx.mori      DEELLDVISWWQQ                                                  192
D.plexippus      DEELLDVISWWQQ                                                  189
H.armigera       DEELLDVISWWQQ                                                  192
S.litura         DEELLDVISWWQQ                                                  192
Consensus        deelldviswwqq
```

Supplementary Fig. S1. Multiple alignment of ash1 gene. Sequences included in analysis are: *Plutella xylostella* (AIZ67914.1), *Bombyx mori* (NP_001037416.1), *Danaus plexippus* (OWR43025.1), *Helicoverpa armigera* (XP_021183890.1) and *Spodoptera litura* (XP_022829400.1). HLH domain is marked with an overbar (55-116 aa)**.**

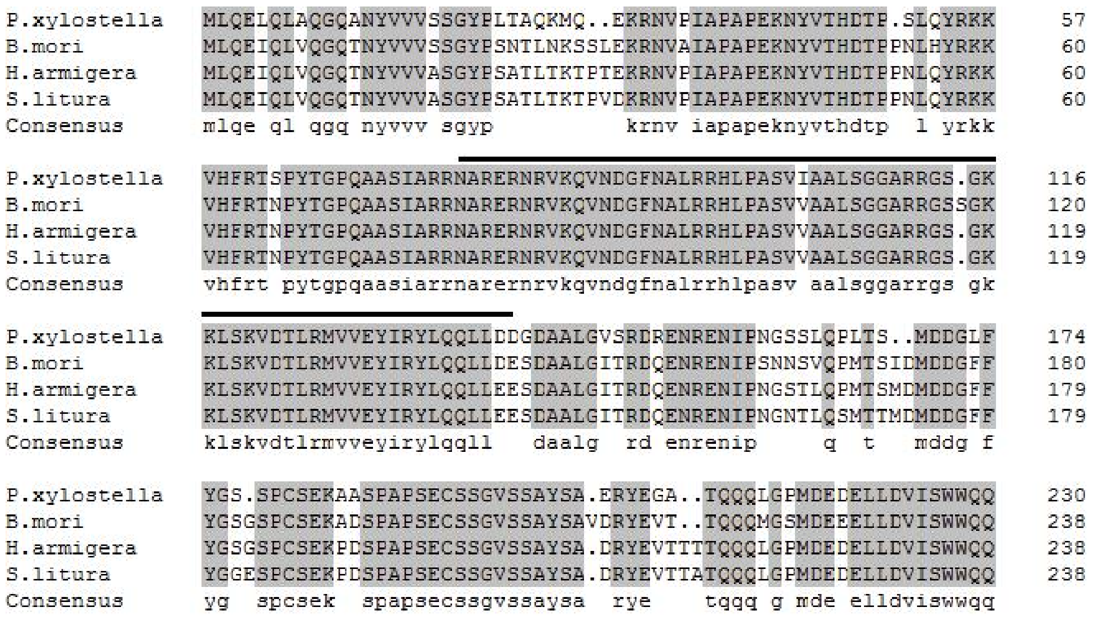

Supplementary Fig. S2. Multiple alignment of ash2 gene. Sequences included in analysis are: *Plutella xylostella* (AIZ67913.1), *Bombyx mori* (NP_001098692.1), *Helicoverpa armigera* (XP_021183904.1) and *Spodoptera litura* (XP_022829477.1). HLH domain is marked with an overbar (77-140 aa).

```
P.xylostella   MLHEMQLAKPAEYFVSQRPHFDAPKMVPIAPAPDKRCLTLDLNVDRRKYNYKNTPYTGVQ    60
B.mori         .....................DSPKMVAIAPAPET...RLTLELEPKKYNYKNCSHNGTQ    36
D.plexippus    .....................EIPKFVPIAPMVDQ...EMDCTVR..KYNYKNNSG...Q    31
O.brumata      ..............VHQKSDYYSQRMVPIAPTPEK..FSLDLQDN..KYTYKSTGYNGAQ    42
Consensus                                v iap                ky yk       q

P.xylostella   SVSTARRNARERNRVKQVNDGFNALRKRLPAAVIAALSGGARRGSGKKLSKVDTLRMVVE   120
B.mori         AASIARRNARERNRVKQVNDGFNALRKRLPAAVVAALSGGARRGSGKKLSKVDTLRMVVE    96
D.plexippus    AASIARRNARERNRVKQVNDGFNALRKRLPAAVVNALSGGARRGSGKKLSKVDTLRMVVE    91
O.brumata      VASIARRNARERNRVKQVNDGFNALRKKLPAAVIAAMAGGSRRGSGKKLSKVDTLKMVVE   102
Consensus        s arrnarernrvkqvndgfnalrk lpaav  a  gg rrgsgkklskvdtl mvve

P.xylostella   YIRHLEGLIEESDEALGIPKQATQVSSMEAYHQDMDEGVFAGRVSPYSDSVPSPVDSECS   180
B.mori         YIRYLQNMIDESDAALGIPKQPSIDLS..TISYEADDGVFER.SSPYTDSVPSPAGSESS   153
D.plexippus    YIKYLENLIDESDASLGVTKEP.MDTS..GI....DEGIFGR.TSPYSDSVPSPANSECS   143
O.brumata      YIKYMENLIEDNDTSHGYSKTVTAT....HFEKDIDEGVYSG.ESPYSVSVPSPL.....   152
Consensus      yi      i   d   g  k               d g      spy  svpsp

P.xylostella   SGVSSAYSGGSNYFINSLQTSTEQHSPMEDDELLDAISWWQH                     222
B.mori         SGVSSNYS.....QGYIPNFQIEEQITPMDDDLLNTISWWQE                     190
D.plexippus    SGVSSSYSN.DHYQINTHNNYYSEVNAVNDSELLDAITWWQQ                     184
O.brumata      ..........................................                     152
Consensus
```

Supplementary Fig. S3. Multiple alignment of ash3 gene. Sequences included in analysis are: *Plutella xylostella* (ALC76152.1), *Bombyx mori* (NP_001098694.1), *Danaus plexippus* (OWR43023.1) and *Operophtera brumata* (KOB69293.1). HLH domain is marked with an overbar (68-131 aa).

```
P.xylostella     MSSIGVVVFRNSPLARAQVLQETVNNSANIANNDVSKNVKREIVILRKKQKLQSP.PTVS     59
B.mori           MSSIDIVVFRNASVNKAQILQETVNNSLNITNNDPNQNARREIIVLRKKQRIQPA.DTVS     59
D.plexippus      MSSIGVVVFRNSPLK.QQVLQESVNNSVNISNND...NVRREIIILRKKQKHQSQ.NTVS     55
H.armigera       MSSIGVVVFRNSPLGKSQVLQESVNNTVNITNNDANQNSTREIIIVRKKPKFQSVPATVS     60
P.rapae          MSSIGVVVFRNSPLK.PQVLQESVNNSVNITNND...NVRREIVIVRKKQKLQS..NTVS     54
Consensus        mssi  vvfrn      q lqe vnn  ni nnd   n  rei   rkk   q    tvs

P.xylostella     VTSLVRAAES..SGPPAAKR..LREE..AEEQARSPTPLAVARRNARERNRVRQVNDGFA    113
B.mori           VPALMRTIEP.SSSSVLAKKARYRENTSSDETVRTPTPLAVARRNARERNRVRQVNDGFA    118
D.plexippus      VTSLVSASEP..NNIVLAKRPKLREN.IPDESTRTPTPLAVARRNARERNRVRQVNDGFA    112
H.armigera       VTSLVRAAEPVTSSAPSAKKSRPNEN.SAEESVRSPTPLAVARRNARERNRVRQVNDGFA    119
P.rapae          VSSLVNTVE...NNNGFAKRPKYRENTNVAEDARSATPLAVARRNARERNRVRQVNDGFA    111
Consensus        v  l    e         ak      e      e  r  tplavarrnarernrvrqvndgfa

P.xylostella     ALRRHIPEEVASAFENANSNRGPNKKLSKVETLRMAVEYIRNLETLLNIGHLDKENNMRP    173
B.mori           ALRRHIPEEVAAAFETTNSNRGPNKKLSKVETLRMAVEYIRNLESLLNIGHADKENTSRS    178
D.plexippus      ALRRHIPDEVAAAFENANSNRGPNKKLSKVETLRMAVEYIRNLENLLNIGHGDKENMSRP    172
H.armigera       ALRRHIPEEVASAFENANSNRGANKKLSKVETLRMAVEYIRNLENLLNIGH.DKENTSRP    178
P.rapae          ALRRHIPEEVAAAFENANSNRGPNKKLSKVETLRMAVEYIRNLESLLNIGHTDKENLSNP    171
Consensus        alrrhip eva afe  nsnrg nkklskvetlrmaveyirnle llnigh dken

P.xylostella     SMESFPSPASSSPRDNSQERSYFCLNSPAMDDDDADEDELDGSMTHMSQQQYVTLPAPDS    233
B.mori           CMESFPSPASSSPRENSQERSYFMISSPALEEEELDEDEIDGL.PGLRQQQYVDIAASEN    237
D.plexippus      SMESFPSPASSSPRDNSQERSYYSLNSPALDDEDMEEDDLDSSLHRLPTQQYMDLP.SET    231
H.armigera       SMESFPSPASSSPRENSQERSYYIIHSPAGDDDDMDEDEIDGS.HHLRRQQYVDLQGTEQ    237
P.rapae          SMESFPSPASSSPRENSQERSYFCLNSPALEDEDLEEDDLDSSLHKISAQQYVDLQASEA    231
Consensus         mesfpspassspr nsqersy    spa       ed  d        qqy

P.xylostella     FQLVSTPHLYEEEDGG.PPMTPSSDLLAHEDVNSHLMDSHFQFPNSAEQFTVIPEQSFCG    292
B.mori           FHLVSTPHLYDEEEGQ..PLTPSSDLLVQDEVNSHILDAHFQFPNSAEHFSVIPEQNYLS    295
D.plexippus      FQLVSTPHLYEEDESR.NPLTPSSDLLGAEEMHSHVLETHFPFPNSAEQFTVIPEQNYC.    289
H.armigera       FQLVATPNLYEEEEGPGQPLTPSSDLLGHEDINPHLMDGHFAFPNSAEQFTVIPERHYLN    297
P.rapae          FQLVSTPRLYEEEDGNGQPLTPSSDLLAQDEMNQHLLDSHFQFPNSAEQFTVIPEQNYCS    291
Consensus        f lv tp ly e       p tpssdll       h    hf fpnsae f vipe

P.xylostella     ESDVMGNESEFEMKYADSLHVQMNHNFNDEE.LPLDAINADMILG..QDQFK..ENTAFL    347
B.mori           ETEVPVSESDFEIKYADSLNQHIQQSFNENT.DIPFALNPDLILPENQFKFKESDNGSFT    354
D.plexippus      EPEAALNDGDFEVKYAETIQN.IHRNFEEDSQLPLEAINPDLMLSHNQYKFK..EESEYL    346
H.armigera       EADVNVNDNDFEVKYSAIMNQQLNHNFTEET..SLPSIDPGLILTHNDYKFKA.EDNSFV    354
P.rapae          ESEVQLSESDFDVKYVDVIHQQIQPNYNEGP..LLDSINSDLLLG..QYKFK..EETPFV    345
Consensus        e         f  ky                          l      fk

P.xylostella     DNDQFSEVELKKELPDI...QVTPEDRAQFEETLKWWQEKTRQARALMKGNH            396
B.mori           ENEDFCEVELKKELPDI...QVTPEDREQFEETLKWWQEKTRQTR.......            396
D.plexippus      DHH.YNEVDLKKELPDI...QVTPEDREQFEETLKWWQEKTRQARPIPKS..            392
H.armigera       EDQFNDDIELKKELPDI...QVTPEDREQFEETLKWWQEKTKQARVLKNNKN            403
P.rapae          EEPPYNGVELKKELPEIHLPEVTPEDREQFEETLKWWQEKTRQARSLSK...            394
Consensus                 lkkelp i    vtpedr qfeetlkwwqekt q r
```

Supplementary Fig. S4. Multiple alignment of *ase* gene. Sequences included in analysis are: *Plutella xylostella* (AIZ67915.1), *Bombyx mori* (NP_001098696.1), *Danaus plexippus* (OWR43026.1), *Helicoverpa armigera* (XP_021183923.1) and *Pieris rapae* (XP_022115661.1). HLH domain is marked with an overbar (98-162 aa).

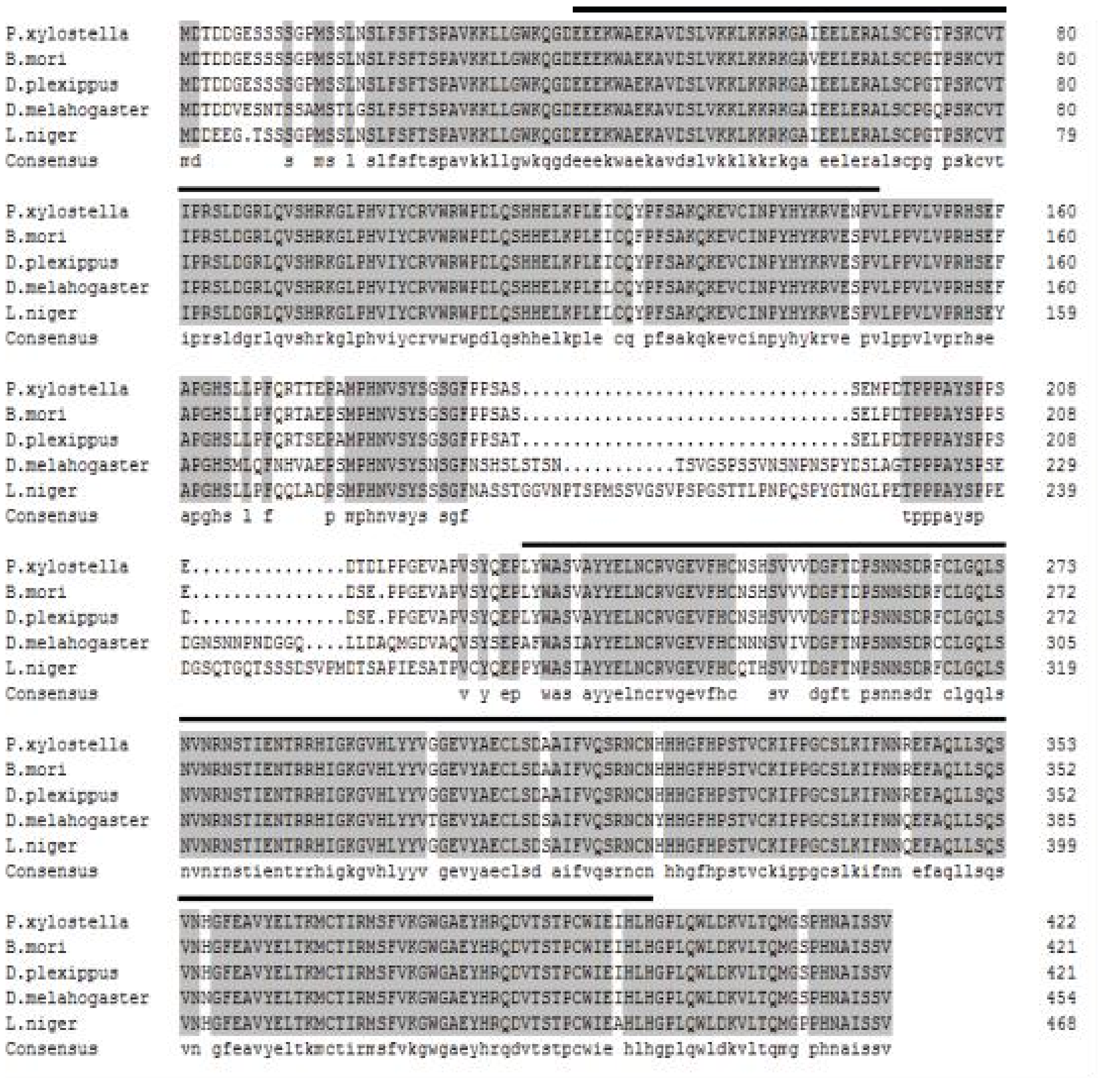

Supplementary Fig. S5. Multiple alignment of *dpp* gene. Sequences included in analysis are: *Plutella xylostella* (ALC76150.2), *Bombyx mori* (XP_004929407.1), *Danaus plexippus* (OWR47416.1), *Drosophila melanogaster* (NP_001259992.1) and *Lasius niger* (KMQ88665.1). DWA and DWB domain is marked with an overbar (39-148 aa, 227-399 aa).

```
P.xylostella    MPCPGPR.GQKRPADQCYDER..PAQTLGMEHCPMPDISDD.YASLQPKK     46
B.mori          ..................................................      0
H.armigera      .PCPMPRAGVKRPSDQCYDER..PPQNVGLDHCPMPDIADDGYASLQPKK     47
Pieris rapae    .PCPVPRSGVKRPSDQCYDER..PSQSLSLEHCGMPDMADDGYASLQPKK     47
S.litura        .PCPMPRAGVKRPSDQCYDER..AQQGVGLEHCPMPDIADDGYASLQPKK     47
T.castaneum     ........GRSLKGDLCYDDRGMPQQGMGLEACGVNDISEDTYNALQPKK     42
Consensus

P.xylostella    SPPSNGKKTKGRVKIKMEYIDNKLRRYTTFSKRKTGIMKKAYELSTLTGT     96
B.mori          ................................RISGVG.EAYELSTLTGT     17
H.armigera      SPPSNGKKTKGRVKIKMEYIDNKLRRYTTFSKRKTGIMKKAYELSTLTGT     97
Pieris rapae    SPPSNGKKTKGRVKIKMEYIDNKLRRYTTFSKRKTGIMKKAYELSTLTGT     97
S.litura        SPPSNGKKTKGRVKIKMEYIDNKLRRYTTFSKRKTGIMKKAYELSTLTGT     97
T.castaneum     SPPSNGKKTKGRVKIKMEYIENKLRRYTTFSKRKTGIMKKAYELSTLTGT     92
Consensus                                       r   g   ayelstltgt

P.xylostella    QVMLLVASETGHVYTFATRKLQPMITSDSGKRLIQTCLNSPDPPQ...TS    143
B.mori          QVMLLVASETGHVYTFATRKLQPMITSDSGKRLIQTCLNSPDPPT...TS     64
H.armigera      QVMLLVASETGHVYTFATRKLQPMITSDSGKRLIQTCLNSPDPPT...TS    144
Pieris rapae    QVMLLVASETGHVYTFATRKLQPMITSDSGKRLIQTCLNSPDPPT...TS    144
S.litura        QVMLLVASETGHVYTFATRKLQPMITSDSGKRLIQTCLNSPDPPT...TS    144
T.castaneum     QVMLLVASETGHVYTFATRKLQPMITSEAGKALIQTCLNSPDPPAGSASG    142
Consensus       qvmllvasetghvytfatrklqpmits  gk liqtclnspdpp

P.xylostella    EQRMAATGFEETELTYNVVDDDMKVRQLAYQSAQYPLEHHPGLAPSPLQQ    193
B.mori          EQRMAATGFEETELTYNVVDEDMKVRQLAYAGTAQYPIEHHPGLAPSPLQ    114
H.armigera      EQRMAATGYEETELTYNVVDEDMK..........................    168
Pieris rapae    EQRMASTGYEETELTYNVVDDDMKV.........................    169
S.litura        EQRMAATGYEETELTYNVVDEDMK..........................    168
T.castaneum     DQRMSATGFEETELTYNISDEDSKVRQMVYGSPHTHTHAH.....SLSGQ    187
Consensus        qrm  tg eeteltyn  d d k

P.xylostella    YHQHP......PCPSPLPLGSLGQPYSHPHLTHPHMSHHPQ             228
B.mori          QYHQHPPCPSPLPLSSLGQPYSHAHLSHPHMSHHPQR....             151
H.armigera      .........................................             168
Pieris rapae    .........................................             169
S.litura        .........................................             168
T.castaneum     GHYSH......LEQSHLGLGGSPQHGGYSQACPSPLP....             218
Consensus
```

Supplementary Fig. S6. Multiple *alignment of srf gene. Sequences included in analysis are: Plutella xylostella (AIZ67912.1), Bombyx mori* (XP_012552250.1), *Helicoverpa armigera* (XP_021190114.1), Pieris rapae (XP_022115280.1), *Spodoptera litura* (XP_022828228.1) and *Tribolium castaneum* (NP_001139383.1).MADS domain is marked with an overbar (56-115 aa).

```
P.Xylostella     MTSQETLVH.HHHLGGSQTPHD...SANSTP.TNVSSKSAFIELQQHGYGPFKGGYQHPHHFGSPVGAGQQNPHEASGFP   75
B.mori           MTSQDNLDH..HHLGGSQTPHDISNSANSTP.TNVS.KSAFIELQQHGYGPFKGGYQHPHHFGSPG..GQQNPHEASGFP   74
M.sexta          MTSQDALDHQHHHLTGSQTPHDIS.SANSTP.TNVSSKSAFIELQQHGYGPFKGGYQHPHHFGSPG..GQQNPHEASGFP   76
P.rapae          MTTQEALEHQHHHLGGTQTPHDISNSANSTP.TNVSSKSAFIELQQHGYG.FKGGYQHPHHFGSPG..GQQNPHEASGFP   76
V.cardui         MTTQE.LDHQHHHLGGSQTPHDISNSTNSTP.TNVSSKSAFIELQQHGYGPFKGGYQHPHHFGSPG..GQQNPHEASGFP   76
D.melanogaster   .........................AASVTPGINIPGKSAFVELQQHAAAGYGGIRSTYQHFGPQG..GQDS.....GFP   48
Consensus                                     tp  n   ksaf elqqh      g      hfg     gq       gfp

P.Xylostella     SPR.SLGYPFPPMHQNTY.GYHLGSYAPQCASPPKDEK..CGLSDDPGLRVNGKGKKMRKPRTIYSSLQLQQLNRRFQRT   151
B.mori           SPR.SLGYPFPPMHQNTY.GYHLGSYAPQCASPPKDEK..CGLSDDPGLRVNGKGKKMRKPRTIYSSLQLQQLNRRFQRT   150
M.sexta          SPR.SLGYPFPPMHQNTY.GYHLGSYAPQCASPPKDEK..CGLSDDPGLRVNGKGKKMRKPRTIYSSLQLQQLNRRFQRT   152
P.rapae          SPR.SLGYPFPPMHQNTY.GYHLGSYAPQCASPPKDEK..CGLSDDPGLRVNGKGKKMRKPRTIYSSLQLQQLNRRFQRT   152
V.cardui         SPR.SLGYPFPPMHQNTY.GYHLGSYAPQCASPPKDEK..CGLSDDPGLRVNGKGKKMRKPRTIYSSLQLQQLNRRFQRT   152
D.melanogaster   SPRSALGYPFPPMHQNSYSGYHLGSYAPPCASPPKDDFSISDKCEDSGLRVNGKGKKMRKPRTIYSSLQLQQLNRRFQRT   128
Consensus        spr  lgypfppmhqn y gyhlgsyap casppkd         d glrvngkgkkmrkprtiysslqlqqlnrrfqrt

P.Xylostella     QYLALPERAELAASLGLTQTQVKIWFQNRRSKYKKMMKAAQVGAPPPGLGLAPGSP...PNNNQLLHGGGGSSSGSQH.S   227
B.mori           QYLALPERAELAASLGLTQTQVKIWFQNRRSKYKKMMKAAQVGAPPPSLGLPPGSP...PSNNQLLHGGGGSSSGSQH.S   226
M.sexta          QYLALPERAELAASLGLTQTQVKIWFQNRRSKYKKMMKAAQV......................................   194
P.rapae          QYLALPERAELAASLGLTQTQVKIWFQNRRSKYKKMMKAAQVGAPPPNLGLPPGSP...PNNNQLLHGGGGSSSGSQH.S   228
V.cardui         QYLALPERAELAASLGLTQTQVKIWFQNRRSKYKKMMKAAQVG.....................................   195
D.melanogaster   QYLALPERAELAASLGLTQTQVKIWFQNRRSKYKKMMKAAQGPGTNSGMPLGGGGPNPGQHSPNQMHSGGNNGGGSNSGS   208
Consensus        qylalperaelaaslgltqtqvkiwfqnrrskykkmmkaaq

P.Xylostella     PSAYQSG.PTQAHSPTPSSTPVSELSPGLSPTGTPWDVKQPQQPNWDVKVGYPRSGEAPDGAKWAAQPPPWH           298
B.mori           PSAYQSGGPTQAHSPTPSSTPVSELSPGLSPTGTHWDVKQPPQASWDVKVGYPTAGRSPDGSSCDVKPP...           295
M.sexta          ........................................................................           194
P.rapae          PSAYQSG.PTQAH...........................................................           240
V.cardui         ........................................................................           195
D.melanogaster   PSHYLPP....GHSPTPSSTPVSELSPEFPPTG....LSPPTQAPWDQKPHW....................           252
Consensus
```

Supplementary Fig. S7. Multiple alignment of dll gene. Sequences included in analysis are: *Plutella xylostella*, *Bombyx mori* (XP_012551909.1), *Manduca sexta* (AAT39558.1), *Pieris rapae* (XP_022120979.1), *Vanessa cardui* (AJS19035.1), Drosophila melanogaster (NP_726486.1)

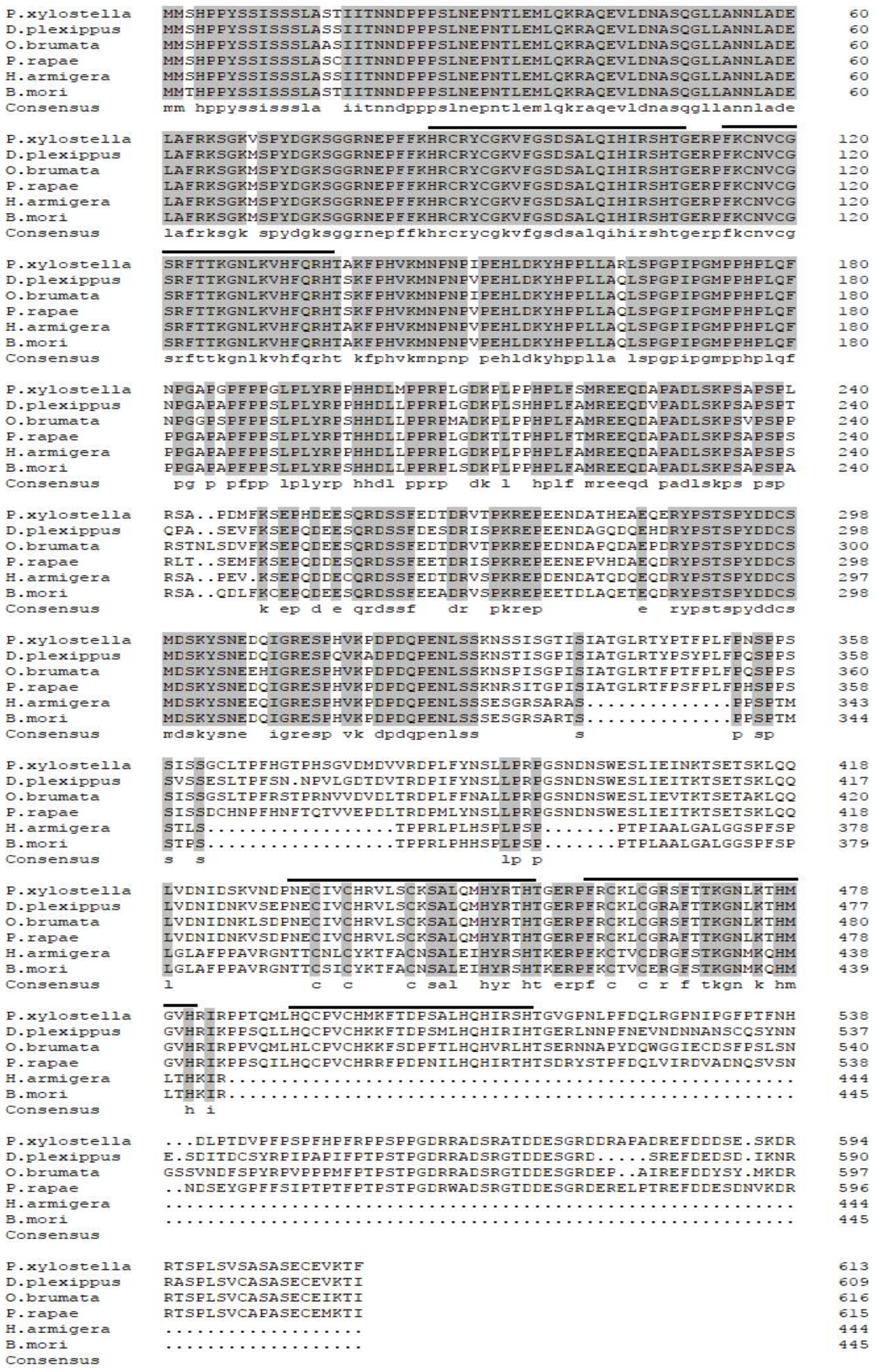

Supplementary Fig. S8. Multiple alignment of salm gene. Sequences included in analysis are: *Plutella xylostella*, (ALC76153.2) *Danaus plexippus* (OWR44146.1), *Operophtera brumata* (KOB70391.1), *Pieris rapae* (XP_022112446.1), *Helicoverpa armigera* (XP_021184031.1), with *Bombyx mori* (XP_012549950.1)

```
P.xylostella   MPHDSYGSYGWAHDHFGPHPAHQGYGQAMPPVPARDTMLPAGPTDGPPSIMSFKAFLAAQ    60
P.rapae        MPHDSYGSYGWAHDHFGPHPAHQGYGQPMPPVPARDAVLPTGPTDGPSTMMSFKAFLAAQ    60
S.litura       MPHDSYG.YGWPHDHFGPHLAHQGYGQPMAPVPARDAVMPLGATDGPPTMMSFKAFLAAQ    59
H.armigera     MPHDSYG.YGWPHDHFSPHPAHQGYGQPMAPVPARDTVLPLGATDGPPTMMSFKAFLAAQ    59
B.mori         MPHDSYGSYGWAHDHFGPHPAHQGYGQPMPPVPARDAVMPVGSAEGPPTMMTFKAFLAAQ    60
P.machaon      MPHDAYGSYGWGHDHFGPHPAHQGYGQPMPPVPARDAVLPMGPSDGPPTMMSFKAFLAAQ    60
C.secundus     ..HDSAA...GGYGSYASSWPHDSYSSQHAYGNHGNNQREMQTAETQPPMMSFKAYLQTQ    55
Consensus        hd                 h  y                         m fka l  q

P.xylostella   DDSITVDDAILKYNEYKLDFRRQQLNEFFVAHKDEEWFKIKYHPEESVKRKEEQLSALKN   120
P.rapae        DDSITVDDAITKYNEYKLDFRRQQLNEFFVAHKDEEWFKIKYHPEESVKRKEEQLAALKN   120
S.litura       DDSITVDDAIQKYNEYKLEFRRQQLNEFFVAHKDEEWFKIKYHPEESIKRKEEQLGALKN   119
H.armigera     DDSITVDDAIQKYNEYKLEFRRQQLNEFFVAHKDEEWFKIKYHPEESVKRKEEQLGALKN   119
B.mori         DDSITVDDAILKYNEYKLEFRRQQLNEFFVAHKDEEWFKIKYHPEESIKRKEEQLSALKN   120
P.machaon      DDSITVDDSIQKYNEYKLEFRRQQLNEFFVAHKDEEWFKIKYHPEESVKRKNEQLAALKN   120
C.secundus     DDNITDDEAIRKYNEYKLEFKRQQLNEFFVTHKEEEWFKIKYHPEDSVKRKDEQMTALKN   115
Consensus      dd it d  i kyneykl f rqqlneffv hk eewfkikyhpe s krk eq  alkn

P.xylostella   RLNVFLELLEQKELDKVTVDVDKSDKLIRLLDTVVIKLEGGTEEDLKILDEPVQEPSS..   178
P.rapae        RLNVFLELLEQGELDKVSVDVDKSDKLIRLLDTVVIKLEGGTEEDLKILDEPAASENTN.   179
S.litura       RLNVFLELLEKGELDKVSVDIDKSDKLIRLLDTVVIKLEGGTEEDLKELDEPPAQENATS   179
H.armigera     RLNVFLELLEKGELDKVSVDIDKSDKLIRLLDTVVIKLEGGTEEDLKELDEPPPQDNVTT   179
B.mori         RLNVFLELLETGELDKVSVDVDKTEKLIRLLDIVVIKLEGGSEEDIKELDEPVNVENSNV   180
P.machaon      RLNVFLELLEQGELDKVTVDVDKSEKLVRLLDTVVIKLEGGTEEDLKALDDPQPQENSTT   180
C.secundus     RVKVFMEFMEQNRLDKVSVDADQSEQLIRLLDAVVIRLEGGTDYDLLALDEPRPATSASG   175
Consensus      r  vf e  e   ldkv vd d    l rlld vvi legg   d   ld p

P.xylostella   ...A.KDKGDADKPVVIDVDAVK.............VKEEKEEDKK..........SEP   210
P.rapae        .NEK.QDKAEPEKAVVIDVDAVK.............VKDEKEDNEK..........IDK   213
S.litura       NDK..ADKTDSDKPVVIDVDDVK.............VKEEKEEEKK.............   210
H.armigera     NDK..ADKLDPDKPVIIDDDDVK.............IKEEKEDNDK.............   210
B.mori         NDK..ADKIVSDKLIVIEDDTVK.............VKEEKEDD...............   209
P.machaon      TEKQ.PEKINTEKPVVIDVDEVK.............VKEEKEKPEE.............   212
C.secundus     QGSAGPGSNQTSKAGTVTAGQVTEKQGSGDAEVKAGSVKEEEEEEPKPFILGEDEDTKKK   235
Consensus                  k        v                k e e

P.xylostella   EKEKKPESP....KPATPLEIEIDPELKQLQEQAKMFSRFN.AA................   249
P.rapae        DKKSDPESP....KPTAPLTMEIDPHLKQLQEQAKLFSRYNTPP................   253
S.litura       EEKKAPESP....KPTAPLTMEIDPHLRQLQEQAKLFSRFNSTG................   250
H.armigera     KEEKKAESP....KPTAPLTMEIDPHLRQLQEQAKLFSRFNSTG................   250
B.mori         KQEKKTESP....KSTAPLTMKIDPHLRHLQEQAKLFSRYNST.................   248
P.machaon      DKDKKAESP....KPTAPLTMEIDPHLRQLQEQAKMFSRFNSQ.................   251
C.secundus     ERDKATESSELKKDPSQPLKLTISSEQVELQKKAKEFLKQKSAEEEDAPMFLFSGQSKKR   295
Consensus            es         pl   i      lq  ak f

P.xylostella   .........GVEEEVK........EPEKEPPPPGS........SGSSSSSSSSSSSSSED   284
P.rapae        .........GTEPDQE........VPEKEPPPPGS........S.SSSSSSSSSSSSSED   287
S.litura       .........TEPEQEE........VKEKEPPPPG.........SSSSSSSSSSSSSSSED   284
H.armigera     .........TEGEPEEKEK.....EKEKEPPPPG.........SSSSSSSSSSSSSSSED   287
B.mori         .........DETEEKPAEK.....PAEKEPPPPG.........SSSSSSSSSSSSSSSED   285
P.machaon      ..........PGSDNEAEK.....PPEKEPPPPAV........SSSSSSSSSSSSSSSED   288
C.secundus     KREYEYESGSESGDEPLAPGDNNGMEEDEPPPPGLEEGKGGDDSYAEDEDKNRSKKMDIS   355
Consensus                                e epppp          s         s

P.xylostella   EGEATTRRKSKSKSKSKTPEKSPARKEKSKSPS..VEKVTENGKE..AEKNEAED.KAES   339
P.rapae        EGES.TRRKSKSKSKSKSPVKSPKAKSRSKSP...VEKIETDSR.....KDKDDD.KSEE   337
S.litura       EGESATRRKSKSKSK..TPDKSPKQQEKAKSKSPSVEKMVTNNIENDKDKEKSGDEKAES   342
H.armigera     EGEATTRRKSKSKSK..TPDKSPKLKEKSKSKSPSVEKISENNNEIDKEKDKSGDEKAES   345
B.mori         EGESTSRRKSKSKSKSKTPDKSPQRKESSKSPSAVKDKPLEN.....KEKNKSGDEKADS   340
P.machaon      EGESATRRKSKSKSK..TPDKSPKRKEKSPSVERIEEEPKAA.....QDKEKTEEEKSEG   341
C.secundus     DDKEDEDEDAKKEDKSDDREDNSDKEGKDKSVKTLAEDGKVDSGDDAEEGVEGDETEREC   415
Consensus                k   k               s

P.xylostella   VI..SDAPEKRETRALHKTTSIFLRNLAPSI                                368
P.rapae        AN..DVEPEKKEVRALHKTTSIFLRNLAPTI                                366
S.litura       IS..DVVMEKKESRALHKTTSIFLRNLAPTI                                371
H.armigera     VS..DVVMEKKETRALHKTTSIFLRNLAPTI                                374
B.mori         VNEVEIVEEKKRTRALHKTTSIFLRNLAPTI                                371
P.machaon      TN..DVIVEKKESRALHKTTSIFLRNLAPSI                                370
C.secundus     KKKRGTSEEAVKPRPLHKTASIFLRNLAPTI                                446
Consensus              e    r lhkt siflrnlap i
```

Supplementary Fig. S9. Multiple alignment of ser gene. Sequences included in analysis are: *Plutella xylostella*, *Pieris rapae* (XP_022130556.1), *Spodoptera litura* (XP_022821559.1), *Helicoverpa armigera* (XP_021188794.1), *Bombyx mori* (XP_012549285.1), *Papilio machaon* (KPJ16753.1), *Cryptotermes secundus* (PNF27227.1).

```
p.xylostella    MKCLCTLVVLTVVVSCQ.ATSKPRRGRGSMWWGIAKAGEPNNLAPMSPGVLYMDPAVHAT     59
S.litura        MKCLWLFVI.VLCFTCE.AANKPRRGRGSMWWGIAKAGEPNNLSPISPGVLYMDPAVHAT     58
P.rapae         ..................AGSKPRRGRGSMWWGIAKAGEPNNLSPLSPGVLYMDPAVHAT     42
B.mori          MKCLWLLVITVLCLRCDTAGTKPRRGRGSMWWGIAKAGEPNNLSPVSPGVLFMDPAVHAT     60
P.xuthus        ................................GIAKAGEPNNISPMSPGVLYMDAGVHST     28
Consensus                                       giakagepnn  p spgvl md  vh t

p.xylostella    LRRKQRRLARENPGALAAVAKGASMAVAECQHQFKNRRWNCSTRNFLRGKNLFGKIVDKG    119
S.litura        LRRKQRRLARENPGVLAAVAKGASMAVAECQHQFKYRRWNCSTRNFLRGKNLFGKIVDRG    118
P.rapae         LRRKQRRLARENPGVLAAVAKGANMAVTECQHQFKYRRWNCSTRNFLRGKNLFGKIVDRG    102
B.mori          LRRKQRRLARENPGVLAAVAK.AQYAFAECQHQFKYRRWNCSTRNFLRGKNLFGKIVDRG    119
P.xuthus        LRRKQRRLAKENPGVLTAVAKGANMAVAECQHQFKYRRWNCTTRNFLRGKNLFGKIVDRG     88
Consensus       lrrkqrrla enpg l avak a  a  ecqhqfk rrwnc trnflrgknlfgkivd g

p.xylostella    CRETAFIYAITSAGVTHAVARACAEGSIESCSCDYSHVGAAPHRARAAAASNVRVWKWGG    179
S.litura        CRETAFIYAITSAGVTHAVARACAEGSIESCTCDYSHIDRVPHRTRAAAAANVRVWKWGG    178
P.rapae         CRETAFIYAITSAGVTHAVSRACAEGSIESCTCDYSHVDRAPHRSRAAAAANVRVWKWGG    162
B.mori          CRETAFIYAITSAGVTHSLARACREASIESCTCDYSHRPRAAQNP.VGGRANVRVWKWGG    178
P.xuthus        CRETAFIYAITSAGVTHAVSRACAEGSIESCTCDYSHLERTPHRTRAAAAANVRVWKWGG    148
Consensus       cretafiyaitsagvth   rac e siesc cdysh              nvrvwkwgg

p.xylostella    CSDNIGFGFRFSREFVDTGERGKTVREKMNLHNNEAGRAHVQTEMRQECKCHGMSGSCTV    239
S.litura        CSDNIGFGFRFSREFVDTGERGKTLREKMNLHNNEAGRAHVQTEMRQECKCHGMSGSCTV    238
P.rapae         CSDNIGYGFKFSREFVDTGERGKTLREKMNLHNNEAGRMHVQTEMRQECKCHGMSGSCTV    222
B.mori          CSDNIGFGFRFSREFVDTGERGKTLREKMNLHNNEAGRRHVQTEMKQECKCHGMSGSCTV    238
P.xuthus        CSDNIGFGFRFSREFVDTGERGKTLREKMNLHNNEAGRAHVQSEMRQECKCHGMSGSCTV    208
Consensus       csdnig gf fsrefvdtgergkt rekmnlhnneagr hvq em qeckchgmsgsctv

p.xylostella    KTCWMRLPSFRSVGDSLKDRFDGASRVQARGGELEAPAQRNDAAPRREPRRDRYRFQLRP    299
S.litura        KTCWMRLPSFRSVGDSLKDRFDGASRVMMPNTDLESPAQRNDAAPHRVPRRDRYRFQLRP    298
P.rapae         KTCWMRLPSFRSVGDSLKDRFDGASRVMVSNTDLETPVQRNDAAPHRVPRRDRYRFQLRP    282
B.mori          KTCWMRLPSFRSVGDSLKDRFDGASRVMLSKADVETPAQRNEAAPHRVPRKDRYRFQLRP    298
P.xuthus        KTCWMRLPSFRSVGDALKDRFDGASRVLMSNSDVETPVLRNDPGAHRVPRRDRYRFQLRP    268
Consensus       ktcwmrlpsfrsvgd lkdrfdgasrv       e p  rn     r pr dryrfqlrp

p.xylostella    SNKEHKAPGVKDLVYVDPSPGFCERNPRLGIAGTHGRACNDSSLGVDGCDLMCCGRGYRT    359
S.litura        HNPDHKSPGAKDLVYLESSPGFCEKNPRLGIPGTHGRACNDTSIGVDGCDLMCCGRGYRT    358
P.rapae         HNPDHKSPGVKDLVYLESSPGFCEKNPRLGIPGTHGRTCNDTSIGVDGCDLMCCGRGYRT    342
B.mori          HNPDHKSPGVKDLVYLESSPGFCEKNPRLGIPGTHGRACNDTSIGVDGCDLMCCGRGYKT    358
P.xuthus        YNPDHKAPGAKDLVYLESSPGFCEKNPRLGIPGTHGRACNDTSIGVDGCDLMCCGRGYKT    328
Consensus        n  hk pg kdlvy   spgfce nprlgi gthgr cnd s gvdgcdlmccgrgy t

p.xylostella    DTMFVYERCNCTFLWCCEVKCKRCHTEK                                    387
S.litura        ETMYVVERCNCTFHWCCEVKCKLCPTEK                                    386
P.rapae         ETMFVVERCNCTFHWCCEVKCKLCRTEK                                    370
B.mori          NTMFVVERCNCTFHWCCEVKCKLCRTEK                                    386
P.xuthus        ETKFVVE.....................                                    335
Consensus        t  v e
```

Supplementary Fig. S10. Multiple alignment of wnt-1 gene. Sequences included in analysis are: *Plutella xylostella*, *Spodoptera litura* (XP_022820536.1), *Pieris rapae* (XP_022116444.1), *Bombyx mori* (NP_001037315.1), *Papilio xuthus* (KPI94016.1)
